# Epigenetic regulation of hematopoietic stem cell fate

**DOI:** 10.1016/j.tcb.2024.08.005

**Published:** 2024-09-12

**Authors:** Yiran Meng, Claus Nerlov

**Affiliations:** MRC Molecular Haematology Unit, MRC Weatherall Institute of Molecular Medicine, University of Oxford, John Radcliffe Hospital, Headington, Oxford, United Kingdom

**Keywords:** Hematopoietic Stem Cell, Fate decision, Epigenome

## Abstract

Hematopoietic stem cells (HSCs) sustain blood cell production throughout the mammalian life span. However, it has become clear that at the single cell level a subset of HSCs is stably biased in their lineage output, and that such heterogeneity may play a key role in physiological processes including ageing and adaptive immunity. Analysis of chromatin accessibility, DNA methylation and histone modifications has revealed that HSC with different lineage bias exhibit distinct epigenetic traits inscribed at poised, lineage-specific enhancers. This allows for lineage priming without initiating lineage-specific gene expression in HSCs, controlling lineage bias while preserving self-renewal and multi-potency. Here we review our current understanding of epigenetic regulation in the establishment and maintenance of HSC fate decisions under different physiological conditions.

## Hematopoietic stem cell heterogeneity

Each day the human blood system generates over 300 billion new cells that underpin hemostasis, oxygen transport, and adaptive as well as innate immunity. The source of these cells is a small pool of long-term self-renewing hematopoietic stem cells (HSCs) that are the cornerstone of the mammalian blood system and play pivotal roles in human health. Through asymmetric division HSCs maintain the stem cell pool while giving rise to differentiating progenitor cells, which simultaneously undergo lineage specification and expansion before differentiating into the >10 mature blood cell types. The central role of HSCs is illustrated by both congenital and acquired HSC deficiencies leading to hematopoietic failure. Furthermore, HSCs are the regenerative cell type in bone marrow transplantation, the most widely used clinical cell therapy with >1.5 million procedures carried out since its inception in 1957 [1]. Understanding how HSCs carry out their homeostatic and regenerative functions, and how this is impacted by ageing, infection and physiological stress, is therefore of considerable interest and clinical importance.

**Figure 1:**
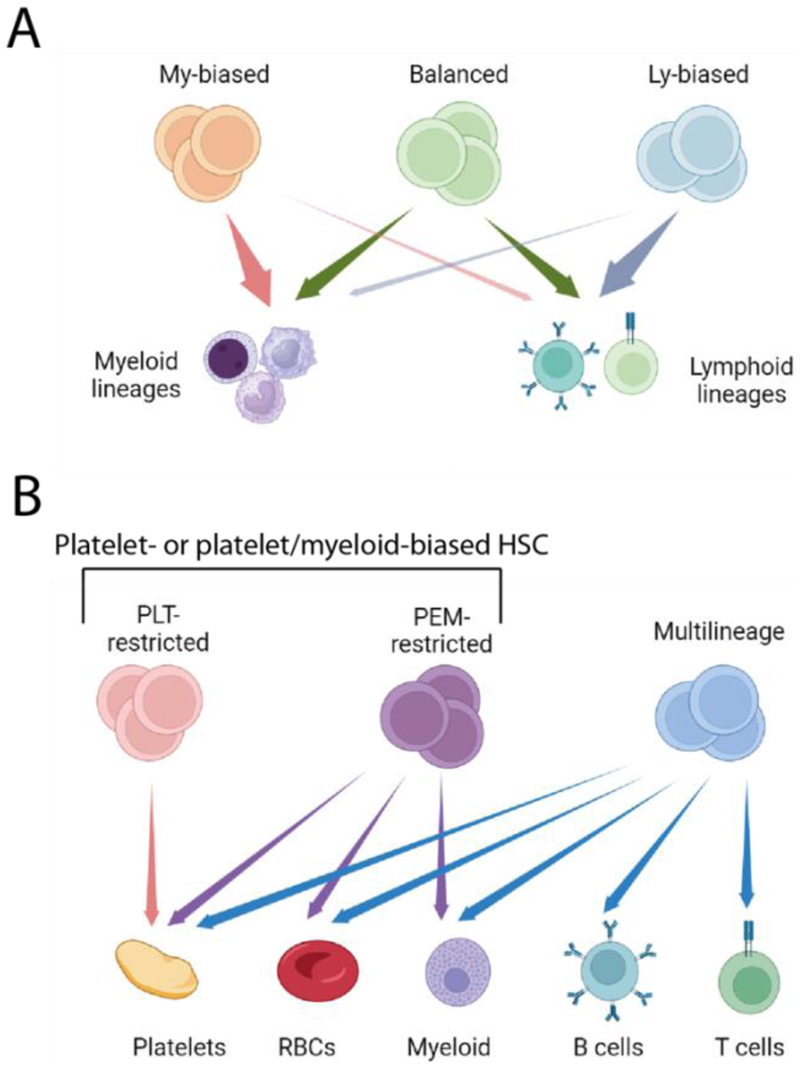
Nomenclature of HSC subtypes. (A) Original HSC subtype nomenclature: Balanced HSCs generate a ratio of myeloid-to-lymphoid output similar to hematopoiesis in general, whereas for My-biased HSCs the ratio is higher, and for Ly-HSCs the ratio is lower. However, this nomenclature does not incorporate erythroid and platelet output, and postulates an HSC subtype (Ly-HSCs) the existence of which is not supported by current experimental evidence. (B) HSC nomenclature based on readout of all peripheral blood cell types, and functional HSC validation. PLT-restricted HSCs only reconstitute platelets after transplantation, PEM-restricted HSCs generate platelets, erythrocytes and myeloid cells, and multilineage HSCs can replenish all five hematopoietic lineages. Together, P- and PEM-HSCs make up platelet-biased HSCs (also often referred to as platelet-myeloid-biased HSCs). This figure is created with BioRender.com.

The cell types produced by HSCs perform a range of critical physiological functions, including blood clotting (platelets) and oxygen transport (erythrocytes). In addition, HSCs produce the cells that compose both the innate (myeloid cells (i.e. granulocytes and monocytes), innate lymphoid cells and natural killer cells) and adaptive immune system (lymphocytes), the latter being critical for the ability to generate immunological memory to new pathogens. HSCs were first identified as cells able to regenerate and sustain all hematopoietic cell types upon transplantation into recipient mice where endogenous hematopoiesis had been ablated by lethal irradiation [2]. Subsequent cell surface marker-based isolation and purification [3] of HSCs was based on identifying cell populations [4] and single cells [5] capable of multi-lineage reconstitution upon transplantation. However, transplantation of HSCs from aged mice at limiting dilution was observed to generate long-term myeloid-only engraftment in some recipients [6], raising the possibility that individual HSCs may have restricted lineage output. This was substantiated by studies transplanting clonal HSC-derived populations [7] and single HSCs [8] where balanced, myeloid-biased and lymphoid-biased engraftment of clonal HSC populations was observed (Fig. 1a). Furthermore, such lineage bias was stable across serial transplantations and shared between daughter HSCs within a clone, indicating that lineage bias is an intrinsic, stable and heritable trait of HSCs. It should be noted that lymphoid-biased engraftment in these studies was propagated by transplantation of total bone marrow cells [7, 8]. However, in these studies lymphoid bias was also associated with loss of serial engraftment activity, and more detailed analysis showed that lymphoid biased clones did not contain detectable HSCs [9]. Currently, it is therefore not clear that HSCs with lymphoid-biased output exist, the more likely hypothesis being that such bias is generated by the persistence of lymphoid cells after exhaustion of multi-potent short-term repopulating cells.

These studies were all based on HSC transplantation, and used allotypes of the CD45 surface marker to detect HSC engraftment. As erythrocytes and platelets do not express CD45 these experiments could not monitor production of erythrocytes and platelets from transplanted HSCs. Furthermore, transplantation involves both microenvironmental perturbation and significant clonal expansion, with the potential to alter HSC function, raising the issue of whether similar lineage bias could be observed outside studies involving HSC transplantation. The first issue was addressed in subsequent studies using transgenic reporter mouse lines to measure platelet and erythrocyte output from single transplanted HSCs, leading to the identification of platelet- and platelet-myeloid biased HSCs [10, 11], and subsequently a set of HSC phenotypes with hierarchically organized fate restriction, with platelet (PLT)-restricted, platelet-erythroid-myeloid (PEM)-restricted and multi-lineage (MUL) HSCs as the main subtypes [9] (Fig. 1b). This classification system is more comprehensive and stringent compared to that based on lineage bias: lineage bias implies higher output of certain lineages compared to others, and can be determined by measuring a subset of lineage outputs. In contrast, fate-restriction is defined based on the absence of detectable output of one or more lineages, and requires comprehensive lineage readout. However, at the molecular level, platelet bias and platelet fate-restriction are similar, and the key distinction to be made is therefore between platelet- (or platelet-myeloid-) biased HSCs, and balanced (or multi-lineage) HSCs [12]. Importantly, studies using transposon-based [13] or recombinase-mediated [14, 15] *in vivo* barcoding were able to identify platelet- and myeloid-biased outputs from single HSCs in unperturbed hematopoiesis, and the molecular signatures of these was highly similar to that identified by comparing clonal platelet-biased and multi-lineage HSCs populations generated by single HSC transplantation [12].

## Regulation of HSC diversity

While these studies firmly established the heterogeneous nature of HSCs, they raised the issue of how HSC lineage bias is molecularly specified and how different HSC subtypes are maintained. An early observation was that ageing altered the lineage output of HSCs in a transplantation setting, with myeloid output increased and lymphoid output decreased in aged HSCs, consistent with intrinsic changes to HSC lineage bias accumulating over time [16]. This was found to correlate with altered composition of HSCs, with ageing increasing the proportion of platelet-myeloid-biased HSCs relative to HSCs with balanced and lymphoid-biased output, as assessed by readout of HSC function at the single cell level [17–20]. This process was observed to be driven by age-related increases in the concentration of inflammatory factors [21–24], a phenomenon frequently referred to as inflammaging, as well as changes in the bone marrow micro-environment [25, 26]. More recently, antibody-mediated depletion of myeloid-biased HSCs was found to increase lymphopoiesis and improve the immune response in aged mice [27]. In addition to highlighting the relevance of HSC heterogeneity to age-related changes in hematopoiesis and immunity, these findings also indicate that HSC subtypes are differentially sensitive to extrinsic signaling, and in particular that intrinsic differences exist that control their responsiveness to inflammatory stimuli. Subtype-specific gene expression patterns have been investigated using surface markers, transgenic reporters [10], single HSC clone analysis [12], and *in vivo* barcoding [14, 28], and by comparing young and aged HSCs [16, 20, 29]. This showed that platelet-myeloid bias, as well as ageing, was associated with increased expression of genes associated with platelet- and myeloid lineage differentiation. In addition, both ageing and platelet-myeloid bias was associated with elevated expression of inflammatory and stemness signatures. Conversely, balanced, multi-lineage output from HSCs was associated with expression of genes involved in cell cycle progression. These observations are consistent with both replicative exhaustion (and associated genomic stress) and differential responsiveness to inflammation underlying the observed shift in HSC subtype abundance. However, beyond transcriptional priming of myeloid and platelet lineages these studies did not identify a molecular basis for how HSC lineage bias is established and maintained, indicating the involvement of additional layers of regulation.

## Epigenetic control of hematopoietic cell fate

**Figure 2:**
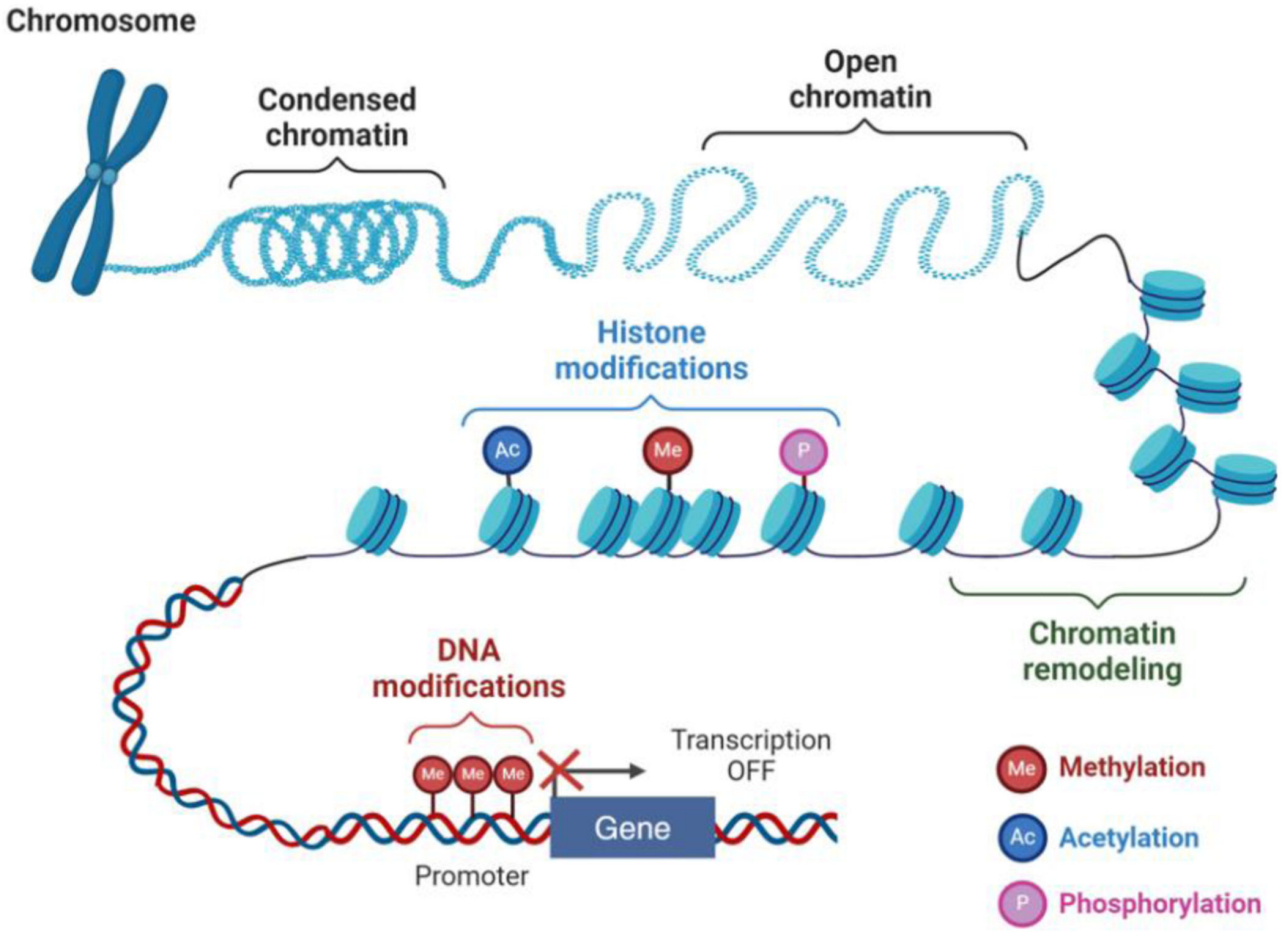
Key types of epigenetic regulation. Chromatin packages DNA and proteins into a compact structure where DNA is wrapped around histones, generating nucleosomes, the basic unit of chromatin. Epigenetic regulators can alter the accessibility of gene regulatory elements through either DNA or histone modification, thereby regulating gene expression. The key DNA modification involves adding a methyl group to cytosine within CpG doublets, generally to suppress transcription. Histone modifications add or remove chemical functional groups, such as methyl, acetyl and phosphate, at specific amino acid residues on histone proteins, to either activate or silence gene expression, depending on the residue and modification. This figure is created with BioRender.com.

Epigenetic modifications, such as DNA methylation and histone modification, can control the accessibility of regulatory elements in the genome, thereby affecting gene expression and subsequent biological processes without altering the core genetic sequence (Figure 2) [30].

DNA methylation involves the addition of a methyl group to the 5 -carbon of cytosine, predominantly in CpG dinucleotides, resulting in 5-methylcytosine (5mC) formation. Around 70-80% of CpG dinucleotides in the mammalian genome are methylated, with CpG clusters associated with gene regulatory regions, termed CpG islands (CGIs) remaining relatively hypomethylated [31]. This modification generally leads to gene silencing by inhibiting the binding of transcription factors to gene regulatory elements or recruiting proteins that compact the chromatin [32]

Histone modifications include various chemical changes to histone proteins, such as acetylation, methylation and phosphorylation. These modifications can either activate or repress gene expression by altering chromatin structure and accessibility. Histone acetylation is the addition of acetyl groups to lysine residues on histone tails, while histone phosphorylation adds phosphate groups to serine, threonine, or tyrosine residues. Both modifications are associated with transcriptional activation. Histone methylation usually involves the addition of methyl groups to lysine or arginine residues and can have variable effects depending on the specific amino acid residue that is modified and the number of methyl groups added [33]. For instance, H3K4me3 (trimethylation of lysine 4 on histone H3) is associated with active transcription, whereas H3K27me3 (trimethylation of lysine 27 on histone H3) is linked to gene repression. Chromatin remodeling refers to an epigenetic process that modifies the structure of chromatin to regulate access to genetic information. This dynamic alteration of chromatin architecture is essential for various cellular processes, including gene expression, and DNA replication and repair. It involves the repositioning, removal, or restructuring of nucleosomes, which are the basic units of chromatin composed of DNA wrapped around histone proteins. These modifications can either condense the chromatin to repress gene expression or relax it to facilitate transcription [34]. Together, these epigenetic mechanisms form a complex regulatory network that mediates gene expression, impacting development and differentiation. Understanding these processes offers insights into the control of gene activity in cell fate decision.

A combination of epigenetic regulators has been shown to ensure that multiple adult stem cell types, including hematopoietic, mammary, intestinal and muscle, are given precise instructions to activate or suppress transcription, maintaining their inherent stemness [35, 36], and dynamic shifts in chromatin play a crucial role in guiding lineage specification, enabling specific transcriptional routes while blocking others, highlights the potent role of epigenetic modifiers in lineage decision making [37]. Epigenetic regulators are also essential players in hematopoiesis [38]. Alterations of chromatin accessibility can induce transcriptional changes in hematopoietic stem and progenitor cells (HSPCs), resulting in an imbalanced production of mature cells [39]. Consequently, techniques that can provide insights into the chromatin landscape, such as the assay for transposase-accessible chromatin using sequencing (ATAC-seq) and chromatin immunoprecipitation assay with sequencing (ChIP-seq), have proven invaluable in understanding the molecular mechanism of hematopoietic fate decision making. Studies using ATAC-seq on HSCs and lineage-committed hematopoietic progenitors, have indicated that chromatin accessibility can in some scenarios identify cell identities more accurately than the transcriptional state [40–42]. This seems to be due primarily to the ability of ATAC-seq to identify lineage-specific cis-regulatory elements that gain accessibility before their corresponding target promoters initiate gene expression. In this manner, chromatin accessibility has the capacity to detect cellular fate decisions before they are transcriptionally executed [43, 44]. Further analysis of the complete hematopoietic hierarchy ranging from HSC to terminally differentiated cells revealed a highly dynamic chromatin landscape throughout hematopoiesis [45, 46]. In HSCs themselves, open cis-regulatory elements (CREs) identified by ATAC-seq are enriched for DNA binding motifs of various lineage-specific transcription factors, indicating epigenetic lineage priming is already present in the HSC compartment. Targeting these open CREs by CRISPR/Cas9-based silencing alters the accessibility to these regulatory regions and allows for epigenetic reprogramming of HSCs [46]. These results are therefore consistent with the differentiation capacity of HSCs being determined at the epigenetic level by chromatin features not readily detectable at the transcriptional level.

## Epigenetic properties of fate-restricted HSCs

**Figure 3:**
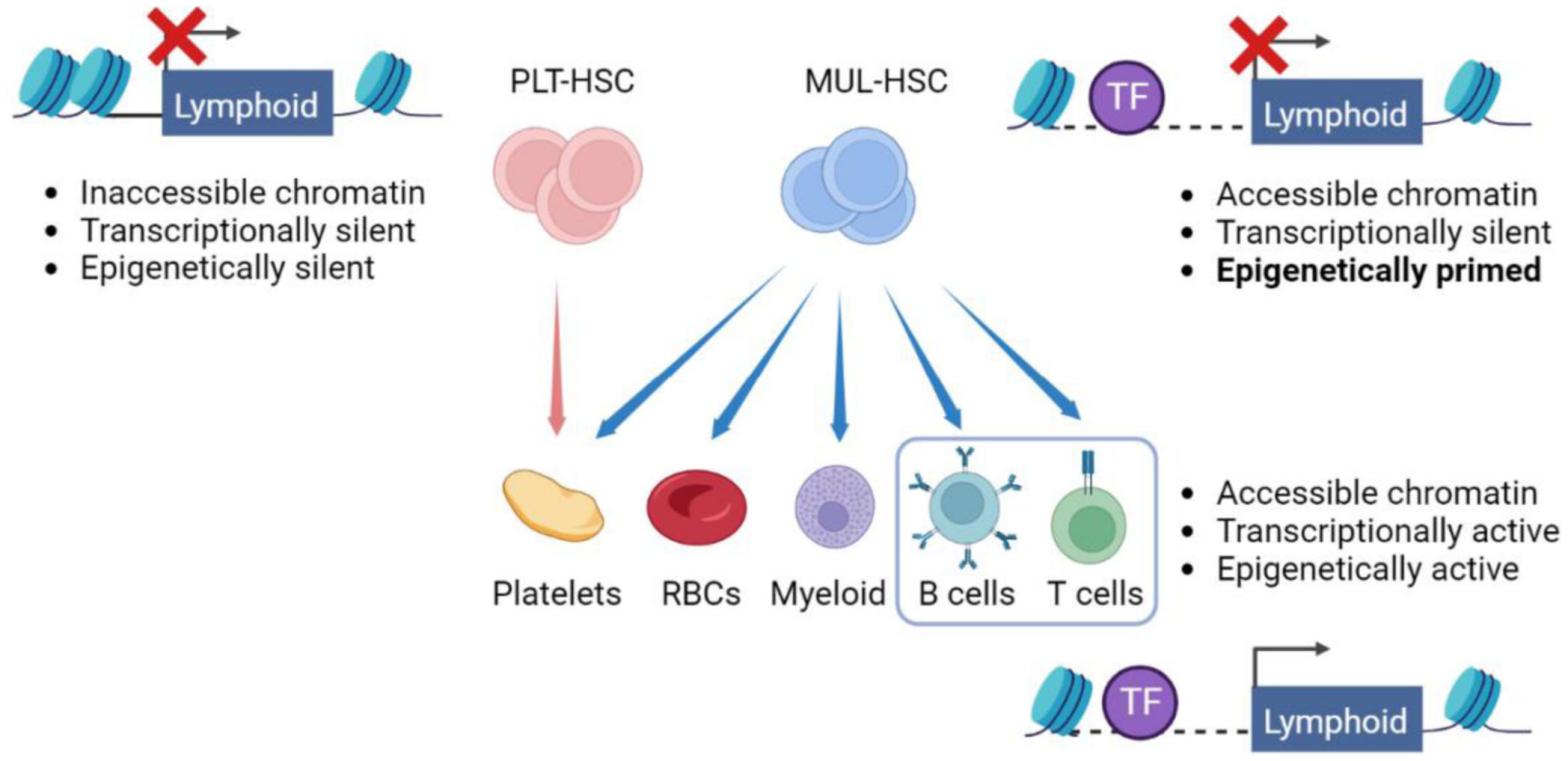
Epigenetic priming of lymphoid fate in HSCs. Lymphoid-specific genes are transcriptionally inactive in both PLT-HSCs and MUL-HSCs. However, chromatin regions associated with lymphoid fate are open and accessible in MUL-HSCs, but not PLT-HSCs, allowing transcription factors to bind to these regulatory elements and epigenetically prime lymphoid differentiation. Upon differentiation MUL-HSCs (but not PLT-HSCs) then activate lymphoid-specific gene expression, leading to the robust production of lymphoid cells. This figure is created with BioRender.com.

The observation that individual HSCs and single HSC-derived clones exhibit distinct and stable differentiation patterns post-transplantation indicates that the forward lineage trajectory of their progeny is intrinsically programmed already at the HSC level. As mentioned above, platelet- and myeloid lineage bias can be detected in HSCs at the transcriptional level. The key difference between platelet/myeloid-biased and balanced (or multi-lineage) HSCs is that only the latter have significant lymphoid output. However, transcriptional lymphoid lineage priming is not detectable in HSCs [20, 47]. While transcriptome analysis therefore does not fully inform us about HSC lineage bias, epigenetic landmarks such as DNA methylation and chromatin accessibility have been found to more accurately predict the behavior of clonal HSC populations [12, 48]. In particular, consistent with these programs being transcriptionally active in HSCs, both platelet- and myeloid lineage-specific chromatin is accessible in HSCs, with the level of accessibility correlating with transcriptional activity and lineage bias [12]. In contrast, while lymphoid cell output does not correlate to transcription of lymphoid lineage-specific genes in HSCs, it has been found to correlate to their epigenetic state: lymphoid-specific chromatin was more accessible in multi-lineage (or balanced) HSCs compared to platelet-biased HSCs, and this was due to increased accessibility of lymphoid enhancers, but not promoters [12]. Similarly, comparison of fluorescently barcoded HSCs clones found a correlation between lymphoid output and decreased DNA methylation of lymphoid enhancers, but again with no difference in promoter methylation [48]. Further analysis showed that lymphoid enhancers were highly enriched for Runx binding sites and showed increased Runx factor occupancy in multi-lineage HSCs [12]. *Runx3* was expressed at higher levels in multi-lineage, compared to platelet-biased HSCs and reinstating Runx3 expression in platelet-biased, aged HSCs increased lymphoid enhancer accessibility. Chromatin accessibility therefore controls HSC lineage bias, both qualitatively and quantitatively, with Runx-family TFs, and Runx3 in particular, a key determinant of lymphoid output through their ability to control the accessibility of lymphoid-specific enhancers (Figure 3).

While epigenetic programming therefore seems to be instrumental in establishing HSC lineage bias it is less clear when during development these epigenetic differences are established. At the functional level, HSCs derived from fetal liver show predominantly balanced, multi-lineage reconstitution, with platelet/myeloid bias becoming prevalent after HSCs migrate to the bone marrow [17]. In contrast, at the molecular level, the *Vwf*-EGFP transgene, which in adult bone marrow is highly expressed in platelet-myeloid biased HSCs, but at much lower levels in multi-lineage HSCs, is expressed uniformly in fetal HSCs, and subsequently lost in a subset of adult bone marrow HSCs [9]. Therefore, HSC diversification seems to be initiated in the bone marrow rather than the fetal liver; however, there is a clear need to investigate this further, to establish the timing and molecular mechanisms involved.

## Epigenetic drift as a hallmark for HSC ageing

In addition to increased platelet-myeloid differentiation bias the aged HSC compartment shows multiple other changes, including increased number and impaired regenerative capacity of phenotypic HSC, and reduced erythrocyte production [16, 20, 24, 25, 49]. Importantly, when aged HSCs are transplanted into young bone marrow environment, their functional impairment persisted [50]. Although the young environment restored transcriptional features of aged HSCs to the young state, the DNA methylation profiles of transplanted, old HSCs remained unaffected by the young micro-environment [51]. In addition, inactivation of the chromatin-associated regulator encoded by the *Phf6* gene can epigenetically reprogram aged HSCs and reverse age-related deficits in a mouse model, consistent with a pivotal role for the epigenome in HSC ageing [52]. The important role of epigenetic regulation in HSC ageing is further supported by several other studies. In particular, comparative analysis of DNA methylation and histone modifications in young and old HSCs has shown that regulatory elements that are linked with proliferation and self-renewal abilities are substantially altered [53–55]. Combined analysis of histone modifications and chromatin accessibility showed an age-related increase of accessible regions that was predominantly associated with enhancer loci [56]. These regions were enriched for binding motifs of transcription factors families (Jun, ATF, STAT, IRF) that are often activated by inflammatory cytokines such as IL-6 and interferons, indicating that the chronic exposure to inflammatory signaling that occurs during physiological ageing induces epigenetic memories in aged HSCs. Finally, consistent with the increased platelet-myeloid lineage bias of aged HSCs, epigenetic analysis also showed that chromatin regions related to these lineages were also more accessible [12]. In contrast, accessibility of regulatory elements linked to lymphoid differentiation decreased, in line with the declining lymphocyte production by aged HSCs. Accordingly, deconvolution of young and aged HSCs using epigenetic signatures of lineage-biased HSC to estimate their clonal composition [12] reproduced the functionally observed increase in frequency of platelet-biased and platelet/myeloid-biased HSC subtypes and concomitant decrease in lymphocyte-producing HSCs in aged mouse bone marrow [19, 20, 57].

## DNA methylation in the regulation of HSC lineage bias

DNA methylation, predominantly occurs at cytosine resulting in 5-methylcytosine (5mC) formation, and is historically linked to heritable silencing of gene expression [32]. The mammalian genome encodes 3 DNA methyltransferases (DNMTs) – DNMT1, DNMT3A, and DNMT3B. Of these DNMT3A and DNMT3B perform *de novo* methylation of unmethylated regions, while DNMT1 maintains existing DNA methylation patterns, particularly during DNA replication [58–60]. While both DNMT1 and DNMT3A/B disruptions lead to hypomethylation, the effects on HSC lineage bias differ. In the mouse, loss of *Dnmt1* function results in a significant bias towards myeloid-erythroid lineage commitment, with a subsequent decline in lymphoid populations [61], whereas *Dnmt3a* loss of function predisposes HSCs towards erythroid differentiation, marked by elevated chromatin accessibility at binding sites for crucial erythroid transcription factors such as *Klf1, Gata1*, and *Tal1* [62]. In addition, *Dnmt3a*–deficient HSCs show highly increased self-renewal capacity, regenerating across 12 or more serial transplantations, in contrast to the poor self-renewal of *Dnmt1*^−/−^ HSCs [63–66]. A plausible link to this prolonged lifespan in *Dnmt3a*^−/−^ HSCs is hypomethylation at the boundaries of extensive unmethylated regulatory regions called canyons. These canyons, roughly 3.5 kb long, are rich in genes associated with stemness, including *Hox* family genes [67], providing a potential mechanism by which self-renewal is enhanced.

Conversely, the Ten-eleven translocation (TET) enzymes oxidize 5mC to 5-hydroxy-methylcytosine (5hmC), leading to DNA demethylation [58]. Among the TET enzymes, TET2’s influence over HSC fate is of most significance. Unlike *Dnmt3a*-deficient HSCs that display erythroid differentiation bias, *Tet2*^−/−^ HSCs show bias towards myelomonocytic differentiation [62, 68]. Analysis on *Tet2*^−/−^ HSCs showed significant disruption of CpG methylation within lineage-specific transcription factor motifs, altering transcriptional priming [62, 68]. Interestingly, despite their divergent influence on HSC lineage bias, both *Dnmt3a*- and *Tet2*-deficient HSCs show enhanced engraftment capacity in a transplantation setting. This suggests that while DNMT3A and TET2, consistent with their opposing roles in sustaining DNA methylation, have opposing roles in determining HSC differentiation bias, they collaborate to limit HSC self-renewal [69], which may require an accurate balance between DNA methylation and -demethylation.

## Epigenetics of clonal hematopoiesis

**Figure 4:**
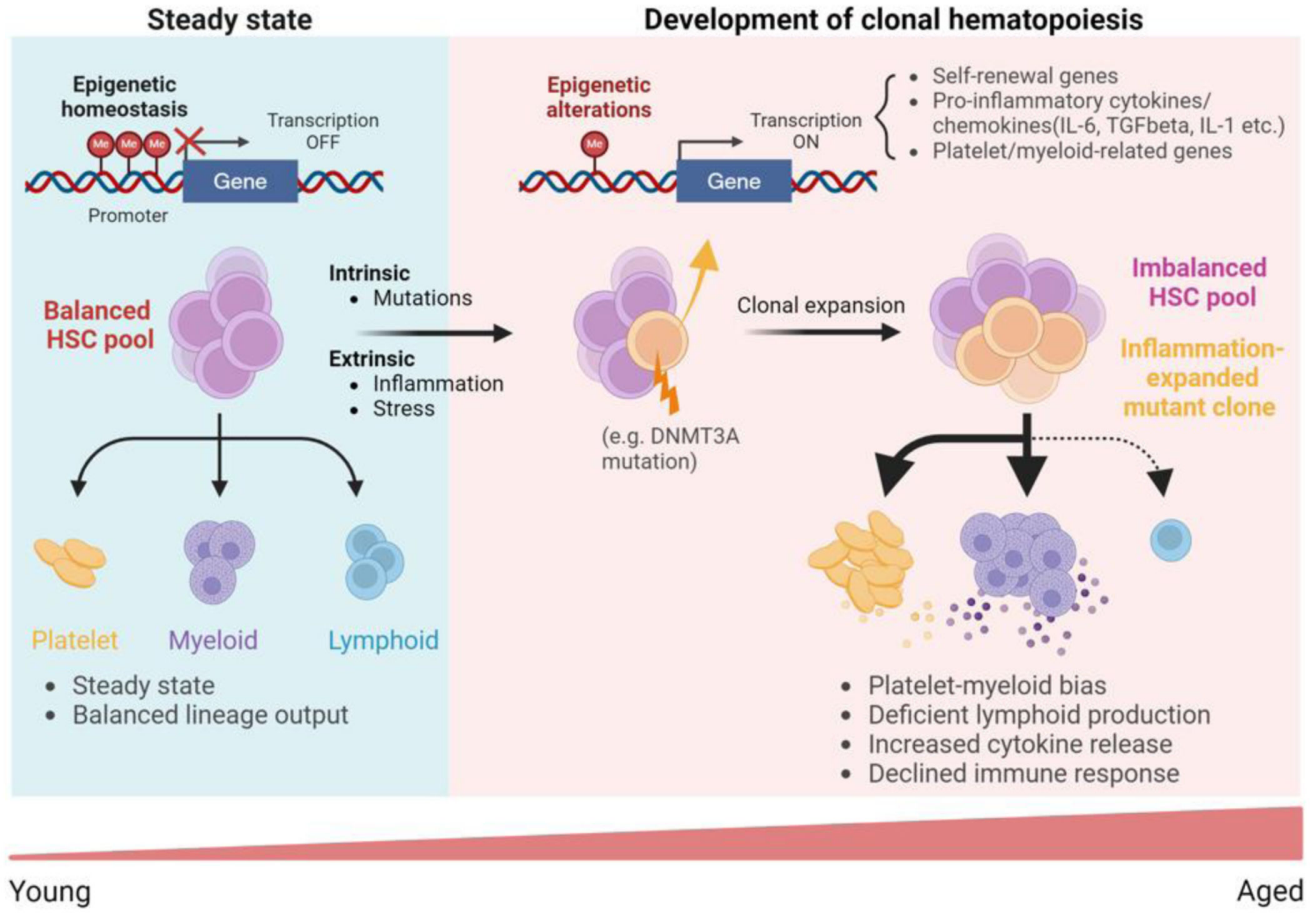
Epigenetic dysregulation in age-related clonal hematopoiesis. In the young steady state hematopoietic lineage output is balanced maintained. During ageing, increased genomic instability causes mutations in HSCs affecting epigenetic regulators, promoting expression of genes associated with self-renewal and inflammation. In addition, an age-associated increase in inflammation generates a micro-environment that selectively expands mutant HSC clones and platelet/myeloid-biased HSCs. Together, these alterations contribute to the development of clonal hematopoiesis and decline in lymphopoiesis. This figure is created with BioRender.com.

In human hematopoiesis, ageing is frequently accompanied by the selective expansion of HSC clones, a phenomenon termed “clonal hematopoiesis” (CH), which leads to overrepresentation of blood cells generated by the expanding clone. Consistent with the observations in genetically modified mice, CH is highly associated with mutations in *DNMT3A* and *TET2* [70, 71], which enhance HSC fitness, as discussed above. Mechanistically, in human HSCs, mutations in *DNMT3A* lead to hypomethylation at genomic sites enriched with binding motifs for crucial hematopoietic transcription factors like MYC and HIF1A [72], increasing chromatin accessibility and consequently upregulating the expression of genes regulated by these transcription factors. These epigenetic alterations modify the trajectory of hematopoietic differentiation, conferring a competitive advantage to the mutant HSC clones. At the same time, HSCs deficient in DNMT3A or TET2 acquire an enhanced immune response to chronic infections, and develop resistance to apoptosis triggered by aging-associated inflammatory signals, including IFNγ, IL-6, and IL-1 [73–76]. A dual mechanism of enhanced fitness – involving both improved stem cell self-renewal and increased resistance to inflammation-induced apoptosis in the aged hematopoietic environment – therefore contributes to the expansion of mutant HSC clones (Figure 4). This may explain the vastly increased prevalence of clonal hematopoiesis in aged individuals, as these mechanisms essentially allow the mutant clones to escape the normal age-associated functional decline of HSCs. These findings also suggest that aged-related phenotypes of HSCs have a significant epigenetic component, making epigenetic regulators a potential target for therapeutic intervention and hematopoietic rejuvenation.

## Epigenetic immune memory in HSCs

Immunological memory, or adaptive immunity, involves the ability to more rapidly respond to previously encountered pathogens. This is traditionally considered a capacity restricted to pathogen-specific memory B and T lymphocytes. However, recent work has shown that innate immune cells, including macrophages, monocytes, and natural killer cells, can also develop a form of memory termed "trained immunity". Upon initial immune challenge, such as microbial infection, these cells undergo reprogramming that enhances their response to subsequent challenges, with a key difference being that such immunity, rather than being strictly pathogen-specific, shows cross-reactivity with other pathogens [77–79]. Furthermore, several recent studies have now extended this principle to hematopoietic stem- and progenitor cells, where microbial antigens have been found to induce immunological memory.

Macrophages play a crucial role in the immune response against *Mycobacterium tuberculosis* (Mtb) infection [80–82]. The Bacillus Calmette–Guérin (BCG), an attenuated form of *Mycobacterium bovis*, has been used to immunize against tuberculosis for more than a century and recent work has shown that this bacteria is capable of inducing trained immunity that boosts monocyte-macrophage production at the expense of lymphoid cell production: intravenous (but not subcutaneous) administration of BCG leads to expansion, as well as transcriptional and functional reprogramming, of the HSPC compartment, promoting myelopoiesis at the expense of lymphopoiesis. Epigenetic analysis of trained HSCs using ChIP-seq revealed that enhancers of genes associated with immune response against BCG were reprogrammed to a poised/active status, thereby priming HSCs to a state that can preferentially and rapidly generate macrophages against Mtb infection upon secondary challenge [83]. This form of trained immunity has been reported in humans as well [84]. That trained immunity enhances resistance to Mtb is further supported by the observation that Mtb infection itself counteracts the establishment of HSC-based trained immunity [85], indicating that this mechanism limits the ability of Mtb to propagate, and that Mtb has evolved to acquire the capability to evade it.

Similarly, β-glucan, a fungal-derived polysaccharide that has shown promising results in cancer immune therapies, also prompts the trained immunity response, enhancing myeloid cells reactivity to subsequent infections [86–88]. As observed for BCG, the HSC and progenitor pools were expanded upon β-glucan exposure and reprogrammed towards myelopoiesis. Neutrophils from β-glucan-trained mice were found to significantly suppress tumor growth when transferred to naive mice, consistent with a general impact of trained granulopoiesis on immune responses. ATAC-seq analysis further revealed that β-glucan induces epigenetic changes that upregulate chromatin accessibility of genes associated with interleukin-1 (IL-1) and IFN-I signaling, both of which are critical in regulating myelopoiesis [87, 88].

Finally, LPS, a component of the outer cell wall of gram-negative bacteria, has been shown to stimulate the formation of epigenetic memory in HSCs. Acute immune reactions triggered by LPS predisposed HSCs towards myeloid lineage differentiation [89]. Furthermore, mice transplanted with HSCs from LPS-treated mice displayed markedly reduced mortality rates when exposed to an infection, and ATAC-seq analysis revealed that the primary LPS exposure upregulates accessibility of chromatin regions enriched for the binding motif of a myeloid transcription factor, C/EBPβ. Deletion of this factor led to the removal of epigenetic marks associated with LPS response and impaired protection against secondary exposure, indicating that the formation of this trained immunity memory in HSCs largely depends on the epigenetic landmarks regulated by C/EBPβ [89].

This work demonstrates that immunological challenge can induce stable changes to HSC populations that enhance subsequent immune responses to similar pathogens, underscoring the importance of epigenetic regulation in the establishment of trained immunity in HSCs and offering exciting possibilities for future preventive interventions for infectious diseases. Importantly, however, the cellular mechanism underlying these changes remains to be determined. As illustrated in Figure 3, increased myelopoiesis could be due to either expansion of existing HSCs with platelet-myeloid bias, or reprogramming of HSCs to increase their myeloid output, or a combination of both. Here, single cell-level functional and molecular analysis will be required to define the underlying cellular mechanism.

## Concluding remarks

It has become clear that epigenetic regulation is critical for many aspects of HSC biology. In particular, the epigenome has a key role in the establishment of HSC heterogeneity, and in the changes that occur to HSCs during ageing, immune responses and in clonal hematopoiesis. In particular, crosstalk between inflammation and the HSC epigenome both enables trained immunity, and regulates the differential response of HSCs to inflammatory signaling during ageing and CH. While further studies will be required to fully understand this crosstalk, this raises the possibility that the increased platelet-myeloid bias seen during ageing is driven by the same basic mechanism that allows trained immunity. In this context, CH may be seen as the expansion of HSC “super-responders” that have a selective advantage when exposed to infection or chronic inflammation. A better understanding of trained immunity at the HSC level, as discussed above, will be necessary to clarify this issue. Importantly, however, CH is associated with an increased overall risk of infection and sepsis [90] and the function of CH-derived immune cells is therefore likely be blunted, rather than enhanced, even if their production is potentially enhanced by a similar mechanism at the HSC level.

At the cellular level, a key question remains how changes to HSC lineage bias occur. As discussed in the context of trained immunity, this could occur through changes at the individual HSC level, or through selective expansion of existing biased HSC populations. Resolving this issue will require the development and application of subtype-specific fate-mapping tools to the relevant physiological scenarios to determine if ageing or infection can remodel the HSC epigenome. Similarly, the developmental timing and molecular mechanisms underlying the initial diversification of HSCs during development remains elusive [91]. Understanding how lineage fate is molecularly encoded in HSCs, and the role epigenetic modifications play in this process, as well as identification of the specific epigenetic regulators and/or extrinsic signals that initiate HSC diversification, has the potential to pave the way for molecular strategies that can be applied to many physiological processes, including ageing, adaptive immunity and clonal hematopoiesis, where HSC diversity and its epigenetic control play a key role.

## Figures and Tables

**Figure 5:**
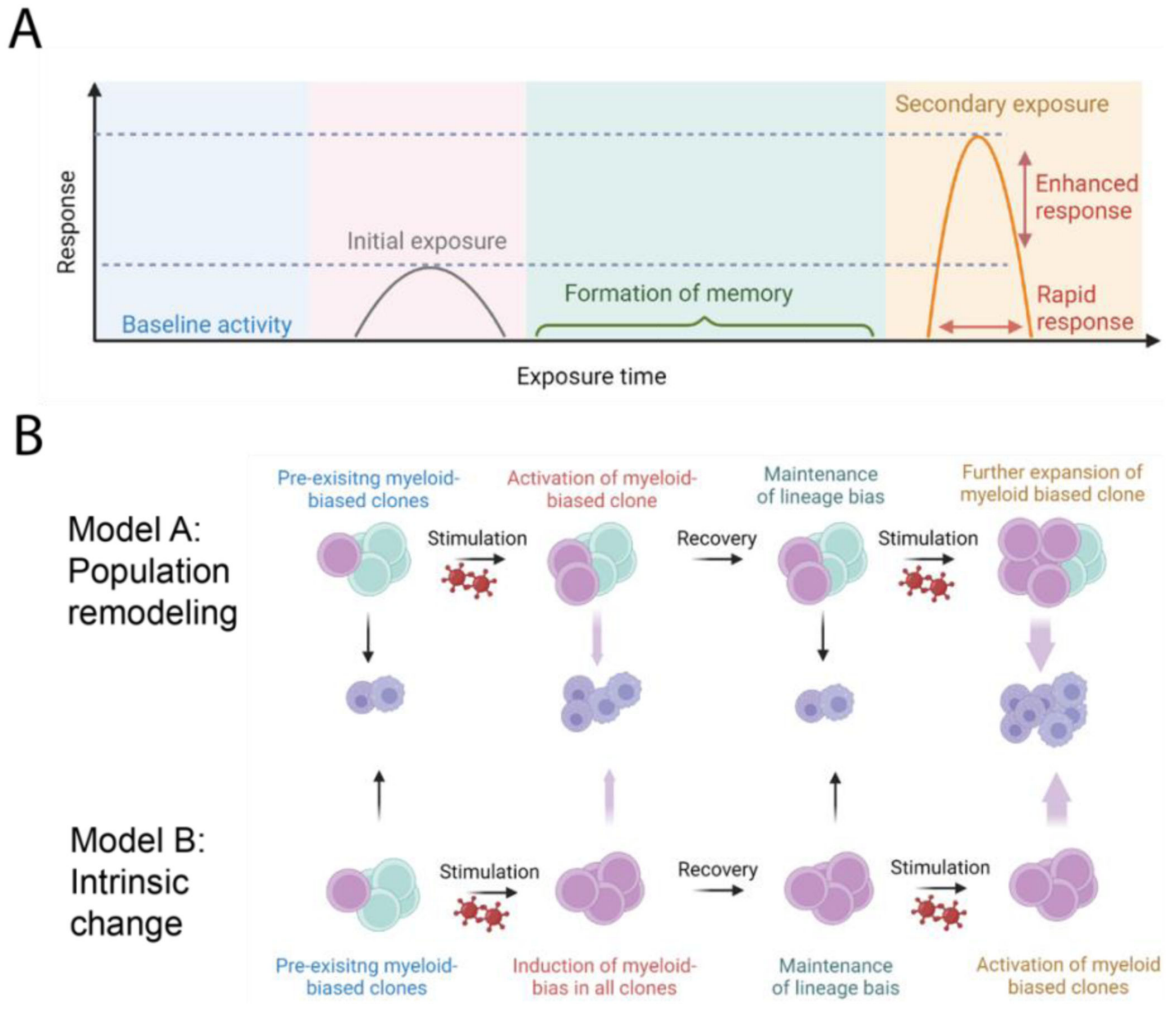
Epigenetic HSCs memory. A) Successive exposures to infections generate an enhanced innate immune response. The formation of epigenetic memory following initial exposure to an infectious agent leads to increased myeloid cell production following a second exposure. B) Two possible models for generation of epigenetic memory. In the first scenario, pre-existing myeloid-biased HSCs are selectively activated and expanded during initial exposure. These expanded clones are maintained after recovery, and can rapidly produce myeloid lineages in response to the secondary exposure. In the second model, primary exposure reprograms HSC clones to an intrinsically more myeloid-biased phenotype, leading to an enhanced differentiation upon secondary exposure. Figure is created with BioRender.com.

## Highlights

(900 characters, including spaces, required; When submitting the manuscript files via Editorial Manager, please upload Highlights as a separate word file using the designated heading)

- The epigenetic landscape varies between individual HSCs with distinct lineage biases.
- Lineage-fate priming can be epigenetically encoded in HSCs prior to initiating lineage-specific gene expression.
- Inflammation and infections induce trained immunity in HSCs via epigenetic memory.
- Disruptions of epigenetic marks link to skewed lineage output and ageing-related phenotypes in HSCs.

## Outstanding questions

(2000 characters, including spaces, required; When submitting the manuscript files via Editorial Manager, please upload the Outstanding Questions Box as a separate word file using the designated heading)

- When is the diversification of HSC heterogeneity first established during hematopoietic development?
- What are the key epigenetic regulators and how do they ensure the establishment and maintenance of HSCs’ fate?
- Can the predetermined lineage fate of HSCs be reprogrammed by (epi)genetic manipulations?
- Can aged HSCs be rejuvenated by reversal of their epigenetic clock?

## Glossary

### Abbreviations

DNA barcode: a short fragment of artificial DNA sequence to label individual cells or molecules
Granulopoiesis: the production of granulocytes
Hematopoiesis: the production of blood cells
Lymphoid-biased HSCs: HSCs that preferentially give rise to lymphocytes over myeloid cells
Myeloid-biased HSCs: HSCs that preferentially give rise to myeloid cells over lymphocytes
Myelopoiesis: the production of myeloid cells
Platelet-restricted HSCs: HSCs that only generate platelets upon transplantation
Platelet/Erythroid-restricted HSCs: HSCs that generate platelets and red blood cells upon transplantation
